# Functional relevance of the multi-drug transporter abcg2 on teriflunomide therapy in an animal model of multiple sclerosis

**DOI:** 10.1186/s12974-019-1677-z

**Published:** 2020-01-08

**Authors:** Lisa Thiele née Schrewe, Kirsten Guse, Silvia Tietz, Jana Remlinger, Seray Demir, Xiomara Pedreiturria, Robert Hoepner, Anke Salmen, Maximilian Pistor, Timothy Turner, Britta Engelhardt, Dirk M. Hermann, Fred Lühder, Stefan Wiese, Andrew Chan

**Affiliations:** 1 Department of Neurology, Inselspital, Bern University Hospital, Department for BioMedical Research (DBMR), University of Bern, Freiburgstrasse, 3010 Bern, Switzerland; 2 Department of Neurology, St. Josef-Hospital, Ruhr-University Bochum, 44801 Bochum, Germany; 3 Sanofi, Cambridge, MA 02142 USA; 4 Theodor Kocher Institute, University of Bern, Bern, Switzerland; 5 Department of Neurology, University of Duisburg-Essen, 45147 Essen, Germany; 6 Institute for Neuroimmunology and Multiple Sclerosis Research, University Medical Center Göttingen, 37075 Göttingen, Germany; 7 Group for Cell Morphology and Molecular Neurobiology, Group of Molecular Cell Biology, Ruhr-University Bochum, 44801 Bochum, Germany

**Keywords:** abcg2, Teriflunomide, Multiple sclerosis, Experimental autoimmune encephalomyelitis

## Abstract

**Background:**

The multi-drug resistance transporter ABCG2, a member of the ATP-binding cassette (ABC) transporter family, mediates the efflux of different immunotherapeutics used in multiple sclerosis (MS), e.g., teriflunomide (teri), cladribine, and mitoxantrone, across cell membranes and organelles. Hence, the modulation of ABCG2 activity could have potential therapeutic implications in MS. In this study, we aimed at investigating the functional impact of abcg2 modulation on teri-induced effects in vitro and in vivo.

**Methods:**

T cells from C57BL/6 J wild-type (wt) and *abcg2*-knockout (KO) mice were treated with teri at different concentrations with/without specific abcg2-inhibitors (Ko143; Fumitremorgin C) and analyzed for intracellular teri concentration (HPLC; LS-MS/MS), T cell apoptosis (annexin V/PI), and proliferation (CSFE). Experimental autoimmune encephalomyelitis (EAE) was induced in C57BL/6J by active immunization with MOG_35–55_/CFA. Teri (10 mg/kg body weight) was given orally once daily after individual disease onset. *abcg2*-mRNA expression (spinal cord, splenic T cells) was analyzed using qRT-PCR.

**Results:**

In vitro, intracellular teri concentration in T cells was 2.5-fold higher in *abcg2*-KO mice than in wt mice. Teri-induced inhibition of T cell proliferation was two fold increased in *abcg2*-KO cells compared to wt cells. T cell apoptosis demonstrated analogous results with 3.1-fold increased apoptosis after pharmacological abcg2-inhibition in wt cells. *abcg2*-mRNA was differentially regulated during different phases of EAE within the central nervous system and peripheral organs. In vivo, at a dosage not efficacious in wt animals, teri treatment ameliorated clinical EAE in *abcg2*-KO mice which was accompanied by higher spinal cord tissue concentrations of teri.

**Conclusion:**

Functional relevance of abcg2 modulation on teri effects in vitro and in vivo warrants further investigation as a potential determinant of interindividual treatment response in MS, with potential implications for other immunotherapies.

## Introduction

The ATP-binding cassette (ABC) transporter family is comprised of molecular transporters that use the energy of ATP hydrolysis to translocate various molecules across extra- and intracellular membranes in essential physiological processes, e.g., nutrient uptake, osmotic homeostasis, and protection from xenotoxins [1]. The transporter ABCG2 is highly expressed at physiological barriers like the intestinal epithelium, the blood-brain barrier (BBB), and hepatocytes. It binds a variety of approved pharmaceutical agents, contributing to their pharmacodynamics and kinetics [2, 3].

Different immunotherapeutics used for the treatment of multiple sclerosis (MS), i.e., teriflunomide (teri), mitoxantrone, and cladribine, are known substrates of ABCG2 [4–7]. Despite increasing treatment options in MS, unmet needs include potential predictors of therapeutic response and adverse drug reactions to possibly guide individualizing treatment strategies [8, 9].

Teri is an oral compound with presumed pleiotropic mechanisms of action. One prominent target appears to be selective inhibition of dihydro-orotate dehydrogenase (DHODH), a key mitochondrial enzyme in the de novo pyrimidine synthesis pathway, leading to a reduction in proliferation of activated T and B lymphocytes [10, 11]. Here, we investigated whether modulation of abcg2 influences teri-induced effects in vitro in T cells and in vivo in experimental autoimmune encephalomyelitis (EAE).

## Materials and methods

### Ethics approval, animal studies, and human samples

Animal experiments were approved by the local authorities (Veterinary Office of the Canton of Bern, Switzerland, no. BE 64/16; North Rhine-Westphalia authorities for animal experimentation, no. 84-02.04.2015.A006). Wild-type (wt) C57BL/6 J (Janvier, France) and *abcg2* knockout (*abcg2*-KO; St. Jude Children’s Research Hospital,Memphis, TN [5];) mice (backcrossed to C57Bl/6 wt mice for > 10 generations) were used for in vivo (i.e., EAE) and in vitro studies. Mice were kept under standardized, pathogen-free conditions. Genotyping was performed with the KAPA Mouse Genotyping Kit (KAPA Biosystems, Woburg, USA); primer: abcg2-KO1 5′-AGG CGA CCT CTT CCA AGA CT-3′, abcg2-KO2 5′-GCA GCG CAT CGC CTT CTA TC-3′, WT1 5′-GTG CCA CCA TGT TCA ACT TA-3′, WT2 5′CTG CCA GAG TAG TGG AAG ATT-3′ (Microsynth, Balgach, Switzerland). Human studies were approved by the local cantonal ethic committee Bern (KEK-BE 2017-00060), and CD3^+^ T cells were isolated from PBMCs of healthy donors.

### Induction, teri treatment, and histopathology of EAE

Chronic EAE was induced by active immunization with 100 μg myelin-oligodendrocyte-glycoprotein peptide 35–55 (MOG_35–55_) (Charité Berlin, Germany) in complete Freund’s adjuvants (CFA) and disease severity was assessed clinically using a 10-point EAE scale: 0, normal; 1, reduced tone of tail; 2, limp tail, impaired righting; 3, absent righting; 4, gait ataxia; 5, mild paraparesis of hind limbs; 6, moderate paraparesis; 7, severe paraparesis or paraplegia; 8, tetraparesis; 9, moribund; 10, death [12]. Teri (provided by Sanofi Genzyme, Cambridge, USA) was dissolved in 25 mM Tris buffer (80 %) in H_2_O (20%) at a pH of 7.5. After individual disease onset (score > 1), mice were treated with teri (10 mg/kg once daily, oral gavage [13, 14];) or respective vehicle for 17–20 consecutive days. Spinal cord tissue was embedded in paraffin and stained with hematoxylin and eosin (H&E) or luxol fast blue (LFB). Immunohistochemistry was performed for CD3^+^ T cells (rat-α-human CD3, 1:100, AbD Serotec, Düsseldorf, Germany), Mac3^+^ macrophages (rat-α-mouse Mac3, 1:100; BD Pharmingen, Heidelberg, Germany), and B220/CD45R^+^ B cells (rat-α-mouse CD45R, 1:200, AbD Serotec). Images were captured with a slide scanner (Pannoramic 250 Flash III, 3DHISTECH, Budapest, Hungary), and the inflammatory score (0, no inflammation; 1, cellular infiltration only in the perivascular areas and meninges; 2, mild cellular infiltration; 3, moderate cellular infiltration [15] or the demyelinated area (ratio of demyelinated area/total area) or number of immune cells within lesions was determined using CaseViewer (3DHISTECH). All in vivo experiments and histological analyses were performed in a blinded setup for the treatment group and genotype of animals.

### FACS analysis of splenic and inguinal lymph node lymphocytes

Lymphocytes from PBS perfused mice suffering from EAE (at day 4 ± 1 after initiation of therapy) or healthy mice were isolated using a Wheaten homogenizer. Subsequently, cells were filtered through a 100-μm nylon mesh and washed (HBSS supplemented with 25 mM HEPES and 5% calf serum), and erythrocytes in splenic samples were lysed. For in vitro teri treatment of splenocytes from healthy mice, cells were incubated with 100 μM teri/DMSO or respective vehicle for 2 h at 37 °C in 5 mL medium (RPMI (Gibco) supplemented with 10% FBS, 2% L-glutamine (Gibco), 1% NEAA (Gibco), 1% sodium pyruvate (Gibco), 1% PenStrep (Gibco), and 0.05 mM β-mercaptoethanol (Merck)). In vitro-treated cells or cells from in vivo-treated mice were stimulated with 50 ng/mL phorbol myristate acetate (PMA; Alexix Biochemicals), 1 mg/mL ionomycin (BioVision, Inc.), and 3.3 μl/5 mL GolgiStop (BD Biosciences) for 4 h at 37 °C in 5 mL medium. After stimulation, cells were collected and stained for CD4, CD25, and FoxP3 using the Treg Detection Kit according to manufacturer’s protocol (Myltenyi) or for intracellular cytokines (IFN-γ, IL-17, GM-CSF, IL-4, IL-10) and surface markers (CD45, CD4, CD8) as adapted from [16]. For surface staining, cells were incubated with primary antibody (Additional file 6: Table S1) mixes in FACS buffer (DPBS, 2.5% FBS, and 0.1% NaN_3_) for 30 min on ice. After a washing step, cells were fixed and permeabilized for 20 min on ice (BD Biosciences; Cytofix/Cytoperm^TM^). Cells were washed and incubated for intracellular staining with primary antibody (Additional file 6: Table S1) mixes in Perm/Wash^TM^ solution (BD Biosciences) for 30 min on ice. Cells were washed twice after the staining and resuspended in FACS buffer. Flow cytometry was performed using an Attune NxT flow cytometer (Thermofisher scientific). Data analysis was performed with FlowJo software.

### Isolation of murine and human T cells and flow cytometry analyses

Isolation of splenic T cells of healthy mice was performed using the Pan T Cell Isolation Kit II according to the manufacturer’s protocol (Miltenyi Biotec, Bergisch Gladbach, Germany). Activated T cells (anti-murine-CD3, 10 μg/mL + anti-murine-CD28, 10 ng/mL; 5% CO_2_, 37 °C [17];) were treated with teri (12.5–100 μM/DMSO, final concentration 0.1%) or DMSO (final concentration 0.1%) as control [18]. After 48 h of incubation, cell proliferation was quantified using the CellTraceTM CFSE (carboxy-flourescein-diacetat-succinimidylester) Cell Proliferation Kit according to the manufacturer’s protocol (Invitrogen, Karlsruhe, Germany; flow cytometry) (Additional file 5: Figure S5A). For pharmacological abcg2 inhibition in wt cells, Fumitremorgin C (FTC, 10 μM) or Ko143 (10 μM) were used. After 48 h of incubation, T cells were analyzed for apoptosis using the FITC annexin V (Anx) Apoptosis Detection Kit I according to manufacturer’s protocol (BD Biosciences, Heidelberg, Germany; flow cytometry) (Additional file 5: Figure S5B). Human T cells (Pan T Cell Isolation Kit (human), Miltenyi Biotec) were stimulated (anti-CD3e, 1 μg/mL, Invitrogen, Karlsruhe, Germany) and treated with teri (50 or 100 μM) with or without FTC (10 μM), and T cells were analyzed for apoptosis after 72 h as described above (Additional file 5: Figure S5B).

### Measurement of teri concentration by HPLC and LC-MS/MS

For teri plasma and tissue concentration, blood and respective organs were collected from PBS-perfused animals after teri-treatment (MOG_35–55_-immunized or non-immunized, 17–20 days after initiation of therapy). Blood was collected by cardiac puncture and plasma was stored at − 80 °C. For teri tissue concentration, the liver, spleen, brain, and spinal cord were frozen in liquid nitrogen and stored at − 80 °C. For intracellular concentrations after in vitro teri treatment, collected cells were diluted in acetonitrile (Bisolve, Valkenswaard, Netherlands) and subsequently lysed using ultrasound. High-performance liquid chromatography (HPLC) followed by liquid chromatography-mass spectrometry (LC-MS/MS) was performed as described previously [19].

### Quantitative real-time PCR analyses

For quantitative real-time PCR (qRT-PCR), healthy animals (Ctrl.) or animals during the acute (16 or 18 days after immunization) or chronic (26 days after immunization) phase were perfused with PBS. The spleen, isolated T cells from the spleen diluted in RLT buffer (Qiagen, Germany), spinal cord tissue, and liver were frozen in liquid nitrogen and stored at − 80 °C. Microvessels from the spinal cord and brain were isolated as previously described [20]. RNA was isolated using TRIZOL followed by the RNAeasy Kit Mini (Qiagen, Germany) and transcribed to cDNA according to the manufacturer’s protocol (Super Mix; Quanta Biosciences, USA). QRT-PCR was performed on an ABI real-time PCR system (Applied Biosystems, Darmstadt, Germany) using PerfeCTa FastMixII master mix (Quanta Bioscience) and abcg2-primer (5′→3′ sense-GCA CCT CAA CCT GCC CAT T; antisense-TCA GGG TGC CCA TCA CAA C; FAM-CAT CTT GAA CCA CAT AAC CTG AAC AGC ATT TG; Microsynth, Baldgach, Switzerland), normalized to the housekeeping gene β-actin (primer: Mm00607939_s1 Actb, Applied Biosystems) using the ΔΔct method.

### Statistical methods

Statistical analyses were performed using GraphPad Prism 7 (GraphPad Software Inc., San Diego, USA). Data is presented as mean ± standard error of the mean (SEM). Statistical tests performed are indicated in the figure legends. Probability level (*p* value) is indicated as *p < 0.05, **p < 0.01, and ***p < 0.001.

## Results

### *abcg2*-deficiency or pharmacological inhibition increases teriflunomide effects in splenic T cells

**Fig. 1:**
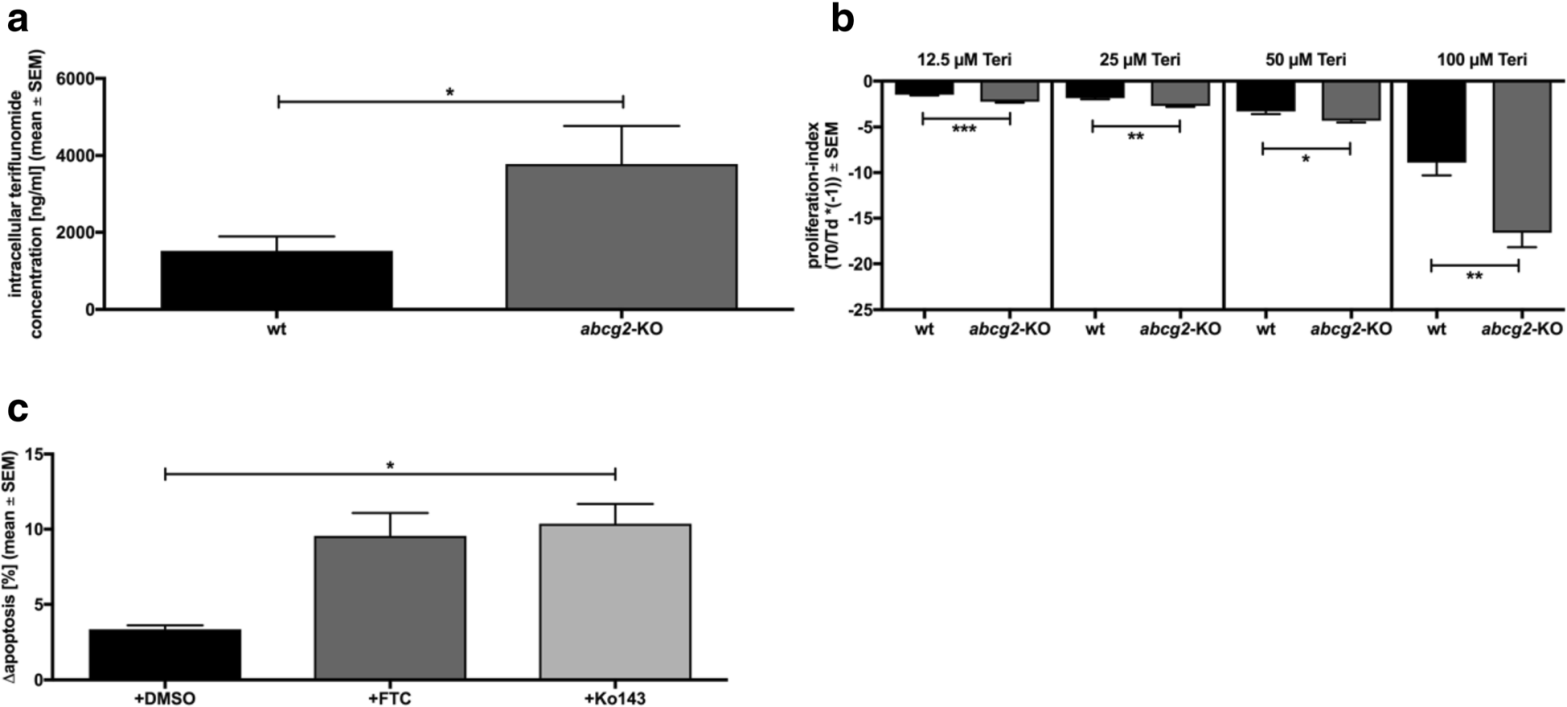
Impact of abcg2-modulation on teri-induced effects in vitro. MACS sorted, activated splenic murine CD3^+^ T cells (α-CD3, 1 μg/mL; α-CD28, 10 ng/mL) from *abcg2*-KO and wt mice were treated with teri (12.5–100 μM) with or without ABCG2 inhibitor (FTC, 10 μM; Ko143, 10 μM). **a** Intracellular teri concentration after 2-6 h incubation; HPLC; LC-MS/MS; *n* = 4, MWU-test: *p < 0.05. **b** Proliferation index after 48 h incubation; CSFE, flow cytometry; n = 6-8, MWU-test: *p < 0.05; **p < 0.01; ***p < 0.001. **c** Apoptosis after 48 h incubation; Anx + PI, flow cytometry; *n* = 3, Wilcoxon test: *p < 0.05. wt: C57BL/6 J wild-type mice; *abcg2*-KO: *abcg2*-deficient mice on C57BL/6 J background. teri, teriflunomide; FTC, Fumitremorgin C

We first investigated whether abcg2 activity determines intracellular teri concentrations. Intracellular teri concentration in splenic T cells from *abcg2*-KO mice was 2.5-fold higher than in T cells from wt animals (*p* < 0.05; Fig. 1a).

To further investigate whether reduced abcg2 activity and increased intracellular teri concentration are associated with functional effects, we analyzed teri-induced inhibition of proliferation as well as apoptosis in splenic CD3^+^ T cells. Increased teri-induced inhibition of T cell proliferation was observed in cells from *abcg2*-KO mice (12.5–100 μM teri, *p* < 0.05; Fig. 1b). Likewise, pharmacological abcg2 inhibition in T cells from wt mice led to an increase of teri-induced apoptosis (Ko143 vs. DMSO: 3.1-fold, *p* < 0.05; FTC vs. DMSO: 2.8-fold, *p* > 0.05; Fig. 1c). In contrast, apoptosis was not increased in human T cells after ABCG2 inhibition (Δ apoptosis, DMSO = 4.8 %; FTC = 5.9%; Wilcoxon test, *p* > 0.05; *n* = 5).

We further evaluated potential immunomodulatory effects of teri on T cell responses in vitro. However, neither in the percentage fractions of CD4^+^CD45^+^ and CD8^+^CD45^+^ T cells nor in the cytokine production (IFN-γ, IL-17, GM-CSF, IL-2, IL-10) relevant differences between genotypes were observed. Only secretion of IL-17 was increased in *abcg2*-KO cells compared to wt cells, however, without differences compared to respective teri-treated cells (Additional file 1: Figure S1).

### *abcg2*-deficency increases the therapeutic response to teriflunomide in experimental autoimmune encephalomyelitis

Next, we investigated whether *abcg2*-mRNA expression is regulated during different phases of EAE in wt mice. Within the spinal cord, *abcg2*-mRNA expression levels decreased during the acute phase (d16 or d18 after immunization; Ctrl. vs. acute: fivefold, *p* > 0.05; Additional file 2: Figure S2A) but increased during the chronic phase (d26 after immunization; Ctrl. vs. acute: twofold, *p* > 0.05; Additional file 2: Figure S2A). Pilot data indicates reduced *abcg2*-expression during the acute phase in microvessels isolated from the spinal cord (Ctrl. vs. acute: threefold, *p* < 0.05, *n* = 4–5, MWU test) but not in brain microvessels (*p* > 0.05, *n* = 2–4, MWU test). In peripheral organs, *abcg2*-expression was significantly decreased in splenic T cells in the acute phase (fivefold, *p* < 0.05; Additional file 2: Figure S2C) but not during the chronic phase. *abcg2*-mRNA expression was also reduced in the liver during the chronic phase (twofold, *p* < 0.05; Additional file 2: Figure S2B).

**Fig. 2:**
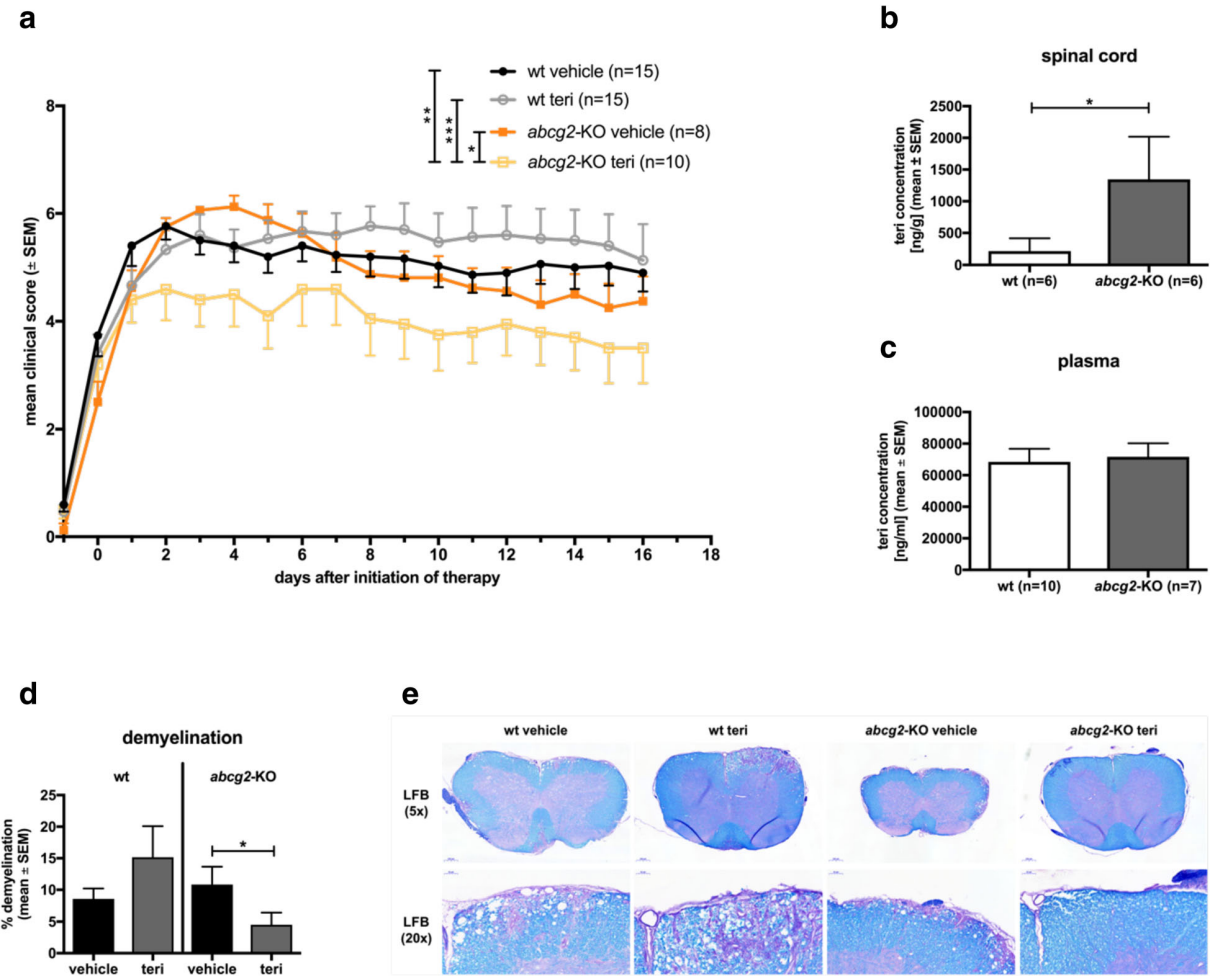
Impact of abcg2-modulation on teri-induced effects in vivo. **a** Teri-treatment (10 mg/kg) of active MOG_35–55_ EAE, once daily p.o., individually after disease onset (score > 1). Data are pooled from three independent experiments with comparable results, each including both genotypes and treatment groups, with a total of 48 mice (9–11 weeks old), number of animals in each group is indicated in the graph. Clinical disease course was assessed using a 10-grade scale; Kruskal-Wallis test. Teri concentration within **b** the spinal cord and **c** plasma after treatment of EAE (10 mg/kg body weight, 17–20 days) during active MOG_35–55_ EAE; HPLC; LS-MS/MS; *n* = 6–10; MWU test. **d** Percentage of demyelination after MOG_35–55_ EAE; luxol fast blue staining (LFB) of spinal cord tissue; *n* = 7–10; MWU test. **e** Representative pictures of LFB staining (× 5 magnification, scale bar 200 μm; × 20 magnification, scale bar 50 μm). The percentage of demyelinated area was calculated as described in the methods. Quantitative results were obtained at two sections of lumbar spinal cord per each mouse. wt: C57BL/6 J wild-type mice; *abcg2*-KO: *abcg2*-deficient mice on C57BL/6 J background. teri, teriflunomide; statistics: *p < 0.05; **p < 0.01; ***p < 0.001

We next investigated whether abcg2 has a functional impact on the therapeutic effects of teri. Teri (10 mg/kg body weight) administered therapeutically after individual disease onset of each animal (score > 1) was not efficacious in wt animals as compared to respective sham-treated controls (mean cumulative EAE score ± SEM; wt teri 5.1 ± 0.3; wt vehicle 4.9 ± 0.3; Fig. 2a). In contrast, using this teri dose, EAE disease course of *abcg2*-KO mice was strongly ameliorated (*abcg2*-KO teri 3.9 ± 0.2; *abcg2*-KO vehicle 4.6 ± 0.3; Fig. 2a). Teri concentration within the spinal cord was significantly increased in *abcg2*-KO mice after treatment (*abcg2*-KO teri vs. wt teri, *p* < 0.05; Fig. 2b). Pilot data further indicate higher teri concentration at similar CD3^+^ T cell numbers in *abcg2*-KO mice in comparison to respective wt animals (0–199 CD3^+^ cells/mm^2^: *abcg2*-KO = 277 ± 186.3 ng/mL, wt = 7.5 ± 7.5 ng/mL; 200–599 CD3^+^ cells/mm^2^: *abcg2*-KO = 2207 ± 1211 ng/mL, wt = 727.5 ± 336.8 ng/mL; mean ± SEM; *n* = 2-3; *p* > 0.05, MWU test). In contrast, teri concentrations in the plasma (*abcg2*-KO teri 71656 ± 8553 ng/ml; wt teri 68472 ± 8300 ng/ml; mean ± SEM, *n* = 7–10, *p* > 0.05, Fig. 2c), spleen (*abcg2*-KO teri 3155 ± 473 ng/g; wt teri 5514 ± 1077 ng/g; mean ± SEM; *n* = 7–8, *p* > 0.05, MWU test), liver (*abcg2*-KO teri 35008 ± 3428 ng/g; wt teri 37,857 ± 3010 ng/g; mean ± SEM; *n* = 6–7, *p* > 0.05, MWU test), and brain (*abcg2*-KO teri 453 ± 95 ng/g; wt teri 495 ± 123 ng/g; mean ± SEM; *n* = 9–10, *p* > 0.05, MWU test) did not show significant differences between *abcg2*-KO mice and wt animals. In non-immunized control animals treated with 10 mg/kg teri for 20 consecutive days, teri concentrations in the spinal cord and plasma were similar (spinal cord, *abcg2*-KO teri = 442 ± 160 ng/g; wt teri = 621 ± 280 ng/g; mean ± SEM, *n* = 3, *p* > 0.999, MWU test and plasma, *abcg2*-KO teri = 63,949 ± 16,544 ng/mL; wt teri = 82,766 ± 7553 ng/mL; mean ± SEM; *n* = 3, *p* > 0.999, MWU test). Lower teri dosages (5 mg/kg and 7.5 mg/kg) did not show beneficial effects on EAE disease course in wt or in *abcg2*-KO mice (data not shown).

Treatment effects of teri on EAE disease course were corroborated histologically by 2.4-fold reduced demyelination of spinal cord tissue in teri-treated *abcg2*-KO mice (*p* < 0.05) whereas teri-treated wt animals did not exhibit a significant difference in comparison to respective sham-treated controls (Fig. 2d, e). During the chronic disease phase, the inflammatory score or number of infiltrating CD3^+^ T cells, Mac3^+^ macrophages, and B220^+^ B cells in the spinal cord did not show statistically significant differences of teri-treated mice compared to vehicle-treated mice within both genotypes; however, observations tended to be consistent with the clinical scores (Additional file 3: Figure S3A–E).

At the peak of EAE (day 4 ± 1 after individual disease onset), peripheral T cell subsets (CD4^+^CD45^+^ T cells, CD8^+^CD45^+^ T cells, and CD4^+^CD25^+^FoxP3^+^ T cells; spleen and inguinal lymph nodes) and cytokine production of T cells (IFN-γ, IL-17, GM-CSF, IL-4, and IL-10) were similar in *abcg2*-KO and wt mice treated with 10 mg/kg teri or vehicle (Additional file 4: Figure S4).

## Discussion

Here, we identify ABCG2 as a determinant of teri-induced effects in a murine model of MS. Increased intracellular teri concentration in T cells lacking *abcg2* indicates that teri is a substrate for active cellular drug efflux in this model. Functional relevance of *abcg2*-deficiency and pharmacological inhibition was shown by increased teri-induced cellular effects on proliferation and apoptosis. In EAE, *abcg2*-mRNA is differentially expressed during the acute and chronic disease stages and in different potential target tissues and cells, and *abcg2*-deficiency leads to increased therapeutic effects accompanied by an increased teri concentration within the spinal cord.

Teri concentrations used in our in vitro experiments were matched to steady-state plasma concentrations of 20–60 mg/L (equivalent to approximately 100–300 μM) reached in humans treated with the approved dose of 7 or 14 mg/day [21]. Intracellular teri concentration was more than twofold higher in *abcg2*-deficient T cells after short incubation time of 2–6 h. Pharmacological inhibition of abcg2 led to an increase of teri-induced apoptosis in murine T cells at the highest teri concentration (100 μM). Using the same concentration, no cytotoxic effects were detected in human lymphocytes, which is in line with previous data [18].

Rodent and human ABCG2 is expressed in most CNS cells with highest expression at physiological barriers like the BBB or blood-spinal-cord barrier [5, 22, 23]. Due to its function at physiological barriers, abcg2 influences the pharmacokinetics of pharmaceutical drugs [1]. This is also likely for teri for which a significant amount was found to be excreted into milk (> 23%) in lactating rats [24] which corresponds to well-known functions of abcg2 contributing to the accumulation of drugs in milk shown in mice [25]. However, although the summary of product characteristics states that teri is a substrate of ABCG2 [7], detailed human data are lacking. Our data indicate that intracellular and tissue levels rather than mere plasma levels are likely to be of relevance. Endogenous substrates of ABC transporters include chemokines and cytokines, suggesting a potential contribution to neuroinflammation and degeneration. Alterations of ABC-transporter activity were demonstrated in active MS-lesions [26]. In addition to inflammatory and oxidative stress [22], apolipoprotein E was also shown to regulate transporter abundancy and localization at the BBB in an experimental stroke model [20]. In peripheral organs, reduced *abcg2*-expression during the chronic phase of EAE was observed in splenic T cells. Also, liver *abcg2*-expression was reduced during EAE, whereas teri plasma levels were not different between genotypes. Our data also indicates that *abcg2*-deficiency alone does not change systemic CNS steady-state teri concentrations which are reached after repeated teri doses in mice after approximately 1 month [24]. Hypothetically, *abcg2*-expression by microvessels and infiltrating (T) cells regulated during EAE may have an impact on teri concentration within the autoimmune inflamed spinal cord. Taken together with our in vitro results, we also hypothesize on *abcg2*-dependent effects on teri efficacy occurring during peripheral T cell proliferation. However, to elucidate the exact site(s) of action of teri and additional impact of abcg2, further experiments including conditional and chimeric animal models are needed as well as experiments on potential functional redundancy. Furthermore, *abcg2*-dependent effects on pleiotropic mechanisms of action of teri (i.e., T cell mitochondrial respiration [11];) with emphasis on teri concentrations within cell organelles would be interesting to investigate. However, these aspects were beyond the scope of the current study which set out to investigate if *abcg2* has an effect on teri-treatment in vivo.

Our findings support a functional relevance of abcg2 in vivo, since a suboptimal teri dosage without clinical effect in wt animals strongly ameliorated the EAE disease course in *abcg2*-KO mice, corroborated by reduced spinal cord demyelination. In addition to mere tissue teri concentrations altered drug kinetics may be relevant. However, whether plasma concentrations in *abcg2*-KO mice increase more rapidly than in wt mice remains to be determined and also tissue concentrations may behave differently. Teri shows strain- and species-dependent differences with higher dosages required for effects on EAE disease onset and/or severity in mice (10 mg/kg/day) than in rats (3–10 mg/kg/day) [13, 14]. In the MOG-induced C57BL/6 EAE model, 10 mg/kg teri administered prophylactically (i.e., before onset of signs) prevents disease occurrence [14]. In MOG_35–55_ EAE in C57BL/6 J wt mice, teri has no effect in a therapeutic setting (i.e., after onset of signs) at tolerable concentrations (teriflunomide Investigator Brochure 2019, internal Sanofi data). In order to address our hypothesis of increased teri effects in *abcg2*-deficient mice, the well-tolerated teri dose which is prophylactically efficacious in MOG-EAE (10 mg/kg, starting on day 3 after immunization [14];) was used in our setting with a therapeutic teri administration after onset of signs. Finally, the dosage used in our experiments is comparable to the human situation with steady-state plasma concentrations in wt mice (68 ± 83 μg/mL) comparable to the steady-state plasma concentrations in humans undergoing therapy with teri with the approved dosages of 7 or 14 mg (20–60 μg/mL) [21].

Different studies suggest that teri has a significant influence on the inflammatory response of various immune cells including reduction of proinflammatory cytokines and modulation of innate immune functions associated with decreased neurotoxicity (e.g., [27, 28]). Our data indicates that in the animal model, therapeutic modulation of teri efficacy in *abcg2*-KO mice is not associated with a modulation of peripheral T cell populations. In MS patients treated with teri and in rodents suffering from EAE treated with the respective prodrug leflunomide, it was demonstrated that different T cell populations were selectively affected with a preferential reduction in Th1 effector cells [11]. The differences observed may be explained by different drugs, treatment duration, or therapeutic setting.

Single nucleotide polymorphisms (SNP) in the *ABCG2* gene can alter protein expression and activity [1]. Both *ABCB1* and *ABCG2* genes are highly polymorphic with a high frequency of functionally relevant variant alleles in different MS populations [5]. Functional relevance for MS treatment was demonstrated with the well-characterized ABCG2 substrate mitoxantrone. In retrospective analyses, association of SNPs in *ABCB1* and *ABCG2* genes with drug effects has been shown at least for relapsing and secondary progressive MS [5] but not for primary progressive MS [29]. During treatment with leflunomide, oral clearance of its active metabolite teri was associated with *ABCG2* SNPs [21].

## Conclusion

Since ABC transporters are highly conserved between rodents and humans [30], our study which demonstrates an impact of *abcg2* on teri-treatment in the animal model of MS argues for further investigations on functional relevance of abcg2-modulation on specific substrates such as teri. Thus far, pharmacological inhibition of respective ABC-transporters as a therapeutic approach has been unsuccessful despite several efforts especially in oncology [2]. Therefore, further investigation of pharmacogenomic association of ABCG2, spatiotemporal regulation under the influence of different drugs, potential redundancy, and functional consequences are needed to substantiate the biologically plausible claim that ABC transporters and their modulation may impact individual treatment responses.

## Supplementary information

**Additional file 1: Figure S1.** Cytokine expression of splenic CD4^+^CD45^+^ and CD8^+^CD45^+^ T cells after in vitro stimulation with teri. Splenocytes from *abcg2*-KO and wt mice were treated with teri (100 μM; 2 h) and assessed for cytokine expression by flow cytometry. (A) Gating strategy to identify CD4^+^CD45^+^ and CD8^+^CD45^+^ T cells. (B) Percentage fraction of CD4^+^CD45^+^ and CD8^+^CD45^+^ cells. Cytokine expression of CD4^+^ T cells (C) and of CD8^+^ T cells (D). Cells isolated from n=6 mice per genotype were divided in 2 treatment groups (DMSO and 100 μM teri). Two-Way ANOVA (Turkey’s multiple comparison test): *p<0.05; **p<0.01; ***p<0.001. wt: C57BL/6J wild type mice; *abcg2*-KO: *abcg2*-deficient mice on C57BL/6J background; teri: teriflunomide.

**Additional file 2: Figure S2.** Differential *abcg2*-expression during experimental autoimmune encephalomyelitis. Relative *abcg2*-mRNA (quantification by TaqMan PCR using ΔΔct method; normalized to *ß-actin*) during the acute or chronic phase of MOG_35-55_ EAE in female C57BL/6 J wild type mice compared to healthy controls. (A) spinal cord, Kruskal-Wallis test; (B) liver, MWU-test; (C) splenic T cells, Kruskal-Wallis test; (D) spleen, MWU-test. Ctrl: healthy control; ac EAE: acute phase of EAE (d16/18 after immunization); chr EAE: chronic phase of EAE (d26 after immunization); statistics: *p<0.05; **p<0.01.

**Additional file 3: Figure S3.** Histolopathological characterization of spinal cords of *abcg2*-KO and wt mice after teri-treatment (10 mg/kg body weight, 17-20 days) during active MOG_35-55_ EAE. (A) Inflammatory score of spinal cord lesions (H&E staining); evaluated as inflammatory score as described in the methods, obtained at two sections of spinal cord tissue per each mouse. (B) Representative pictures of H&E staining (40x magnification, scale bar 20 μm). Quantification of (C) CD3+ cells (T-cells), (D) Mac3+ cells (macrophages) and (E) B220+ cells (B-cells); evaluated as cells/mm^2^, obtained at two sections of spinal cord tissue per each mouse. A-D: n=6-13; MWU-test, p=ns. wt: C57BL/6J wild type mice; *abcg2*-KO: *abcg2*-deficient mice on C57BL/6J background; teri: teriflunomide.

**Additional file 4: Figure S4.** Immunomodulation of T cell responses after teri-treatment during MOG_35-55_ EAE: Cytokine expression CD4^+^CD45^+^ and CD8^+^CD45^+^ T cells from spleen (A-D) and inguinal lymphnodes (E-H) and percentage of CD25^+^FoxP3^+^ of CD4^+^ T cells from spleen and inguinal lymphnodes (I-J). Teri-treatment (10 mg/kg) of active MOG_35-55_ EAE, once daily p.o. (4±1 days), individually after disease onset (Score>1) in *abcg2*-KO and wt mice. (A, E) Gating strategy to identify CD4^+^CD45^+^ and CD8^+^CD45^+^ T cells. (B, F) Percentage fraction of CD4^+^CD45^+^ and CD8^+^CD45^+^ cells. Cytokine expression of CD4^+^ T cells (C, G) and of CD8^+^ T cells (D, H). (I) Gating strategy to identify CD25^+^FoxP3^+^ cells of CD4^+^ cells. (J) Percentage portion of CD4^+^CD25^+^FoxP3^+^ cells. Data were assessed by flow cytometry. Two-Way ANOVA (Turkey’s multiple comparison test): *p<0.05; **p<0.01. wt: C57BL/6 J wild type mice; *abcg2*-KO: *abcg2*-deficient mice on C57BL/6 J background; teri: teriflunomide; LN: inguinal lymphnodes.

**Additional file 5: Figure S5.** Gating strategies of proliferation and apoptosis assays (see Fig. 1). (A) Gating strategy to identify the fraction of proliferating CFSE^+^PI^-^ T cells. Representative figures of wt T cells treated with DMSO or teri (100 μM teri) for 48 h. Proliferation index was calculated as follows: normalized to ∅ cell death ( $=\% Proliferation\times \frac{\left(100-\varnothing cell\ death\right)}{100}$ ); quotient vehicle-treated cells to teri-treated cells ($=\frac{\% proliferation\ (vehicle)}{\% proliferation\ (teri)}$ x (-1)). (B) Gating strategy of apoptosis to identify percentage fraction of apoptotic T cells. Representative picutres of wt cells treated with DMSO for 48 h. Apoptosis was calculated as sum of Anx^+^, AnxPI^+^ and PI^+^ T cells.

**Additional file 6: Table S1.** Flow cytometry antibodies.

## Data Availability

The datasets analyzed during the current study are available from the corresponding author upon reasonable request.

## Abbreviations

ABC: Adenosine triphosphate-binding cassette
Anx: Annexin V
BBB: Blood-brain barrier
CFSE: Carboxy-flourescein-diacetate-succinimidylester
CNS: Central nervous system
Ctr: Control (i.e., healthy or non-immunized mice)
DHODH: Dihydro-orotate dehydrogenase
EAE: Experimental autoimmune encephalomyelitis
FTC: Fumitremorgin C
GM-CSF: Granulocyte macrophage colony-stimulating factor
H&E: Hematoxylin and eosin
HPLC: High-performance liquid chromatography
IFN-γ: Interferon gamma
KO: Knockout
LFB: Luxol fast blue
LS-MS/MS: liquid chromatography-mass spectrometry
MOG: Myelin-oligodendrocyten-glycoprotein
MS: Multiple sclerosis
PBMC: Peripheral blood mononuclear cells
PI: Propidium iodide
qRT-PCR: Quantitative real-time PCR
SNP: Single nucleotide polymorphism
Teri: Teriflunomide
