# Supplementary material for: Functional relevance of the multi-drug transporter abcg2 on teriflunomide therapy in an animal model of multiple sclerosis
Source: J Neuroinflammation. 2020 Jan 8;17:9. doi: 10.1186/s12974-019-1677-z (PMC6951012; doi:10.1186/s12974-019-1677-z)
Supplement: Supplementary file 1 — Additional file 1: Figure S1. Cytokine expression of splenic CD4+CD45+ and CD8+CD45+ T cells after in vitro stimulation with teri. Splenocytes from abcg2-KO and wt mice were treated with teri (100 μM; 2 h) and assessed for cytokine expression by flow cytometry. (A) Gating strategy to identify CD4+CD45+ and CD8+CD45+ T cells. (B) Percentage fraction of CD4+CD45+ and CD8+CD45+ cells. Cytokine expression of CD4+ T cells (C) and of CD8+ T cells (D). Cells isolated from n=6 mice per genotype were divided in 2 treatment groups (DMSO and 100 μM teri). Two-Way ANOVA (Turkey’s multiple comparison test): *p<0.05; **p<0.01; ***p<0.001. wt: C57BL/6J wild type mice; abcg2-KO: abcg2-deficient mice on C57BL/6J background; teri: teriflunomide. [file 12974_2019_1677_MOESM1_ESM.pdf]

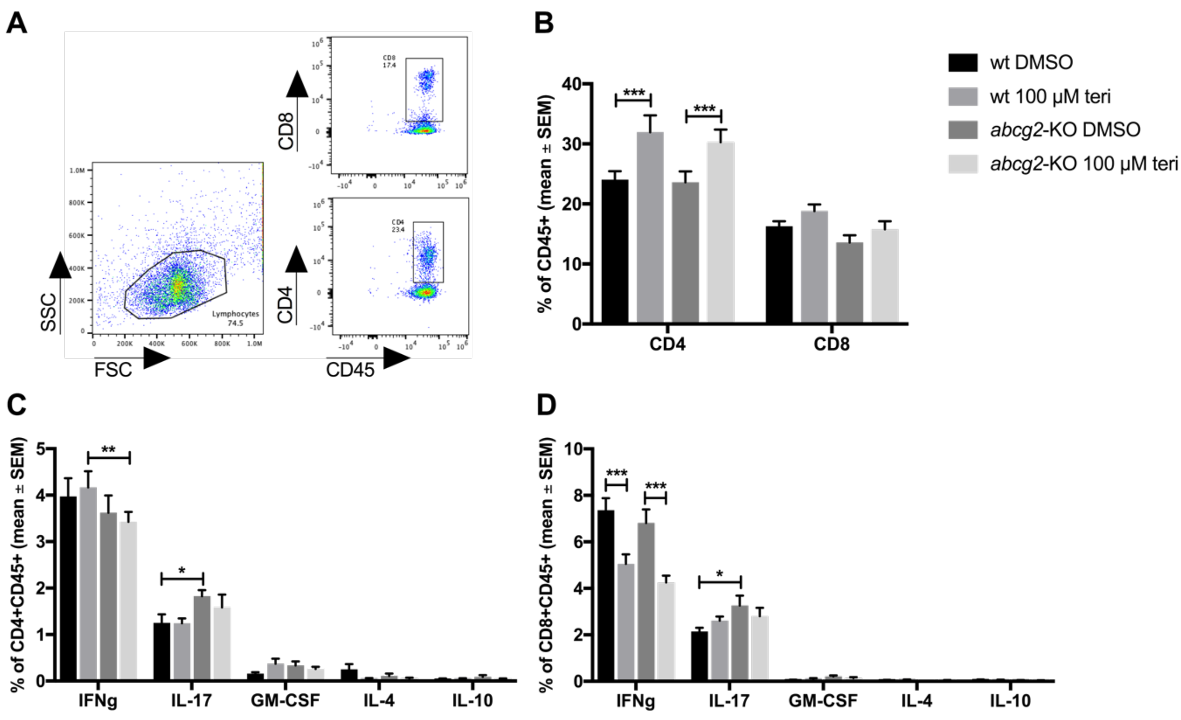

Supplementary Figure 1: Cytokine expression of splenic CD4^{+}CD45^{+} and CD8^{+}CD45^{+} T cells after *in vitro* stimulation with teri. Splenocytes from *abcg2*-KO and wt mice were treated with teri (100 µM; 2h) and assessed for cytokine expression by flow cytometry. **(A)** Gating strategy to identify CD4^{+}CD45^{+} and CD8^{+}CD45^{+} T cells. **(B)** Percentage fraction of CD4^{+}CD45^{+} and CD8^{+}CD45^{+} cells. Cytokine expression of CD4^{+} T cells **(C)** and of CD8^{+} T cells **(D)**. Cells isolated from n=6 mice per genotype were divided in 2 treatment groups (DMSO and 100 µM teri). Two-Way ANOVA (Turkey´s multiple comparison test): *p<0.05; **p<0.01; ***p<0.001. wt: C57BL/6J wild type mice; *abcg2*-KO: *abcg2*-deficient mice on C57BL/6J background; teri: teriflunomide.
