# Supplementary material for: Functional relevance of the multi-drug transporter abcg2 on teriflunomide therapy in an animal model of multiple sclerosis
Source: J Neuroinflammation. 2020 Jan 8;17:9. doi: 10.1186/s12974-019-1677-z (PMC6951012; doi:10.1186/s12974-019-1677-z)
Supplement: Supplementary file 2 — Additional file 2: Figure S2. Differential abcg2-expression during experimental autoimmune encephalomyelitis. Relative abcg2-mRNA (quantification by TaqMan PCR using ΔΔct method; normalized to ß-actin) during the acute or chronic phase of MOG35-55 EAE in female C57BL/6 J wild type mice compared to healthy controls. (A) spinal cord, Kruskal-Wallis test; (B) liver, MWU-test; (C) splenic T cells, Kruskal-Wallis test; (D) spleen, MWU-test. Ctrl: healthy control; ac EAE: acute phase of EAE (d16/18 after immunization); chr EAE: chronic phase of EAE (d26 after immunization); statistics: *p<0.05; **p<0.01. [file 12974_2019_1677_MOESM2_ESM.pdf]

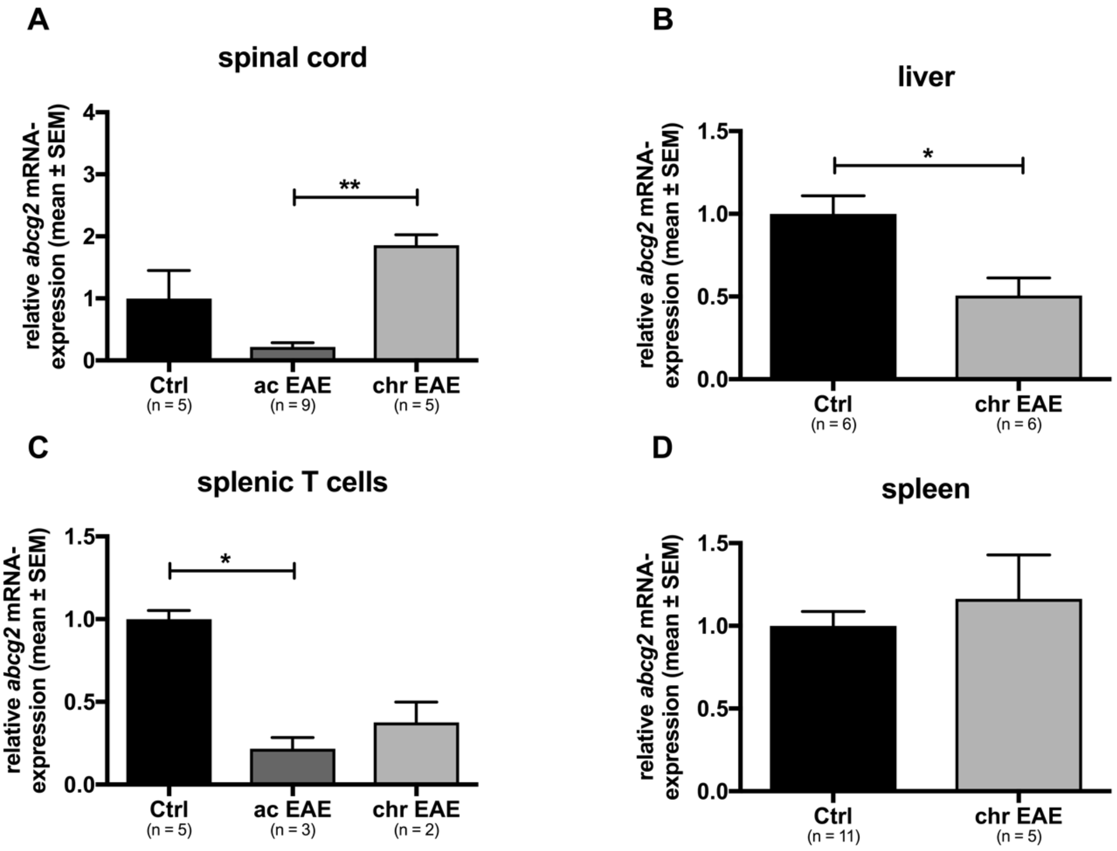

Supplementary Figure 2: Differential *abcg2*-expression during experimental autoimmune encephalomyelitis. Relative *abcg2*-mRNA (quantification by TaqMan PCR using ΔΔct method; normalized to *ß-actin*) during the acute or chronic phase of MOG_{35-55} EAE in female C57BL/6J wild type mice compared to healthy controls. **(A)** spinal cord, Kruskal-Wallis test; **(B)** liver, MWU-test; **(C)** splenic T cells, Kruskal-Wallis test; **(D)** spleen, MWU-test. Ctrl: healthy control; ac EAE: acute phase of EAE (d16/18 after immunization); chr EAE: chronic phase of EAE (d26 after immunization); statistics: *p<0.05; **p<0.01.
