# Supplementary material for: Functional relevance of the multi-drug transporter abcg2 on teriflunomide therapy in an animal model of multiple sclerosis
Source: J Neuroinflammation. 2020 Jan 8;17:9. doi: 10.1186/s12974-019-1677-z (PMC6951012; doi:10.1186/s12974-019-1677-z)
Supplement: Supplementary file 3 — Additional file 3: Figure S3. Histolopathological characterization of spinal cords of abcg2-KO and wt mice after teri-treatment (10 mg/kg body weight, 17-20 days) during active MOG35-55 EAE. (A) Inflammatory score of spinal cord lesions (H&E staining); evaluated as inflammatory score as described in the methods, obtained at two sections of spinal cord tissue per each mouse. (B) Representative pictures of H&E staining (40x magnification, scale bar 20 μm). Quantification of (C) CD3+ cells (T-cells), (D) Mac3+ cells (macrophages) and (E) B220+ cells (B-cells); evaluated as cells/mm2, obtained at two sections of spinal cord tissue per each mouse. A-D: n=6-13; MWU-test, p=ns. wt: C57BL/6J wild type mice; abcg2-KO: abcg2-deficient mice on C57BL/6J background; teri: teriflunomide. [file 12974_2019_1677_MOESM3_ESM.pdf]

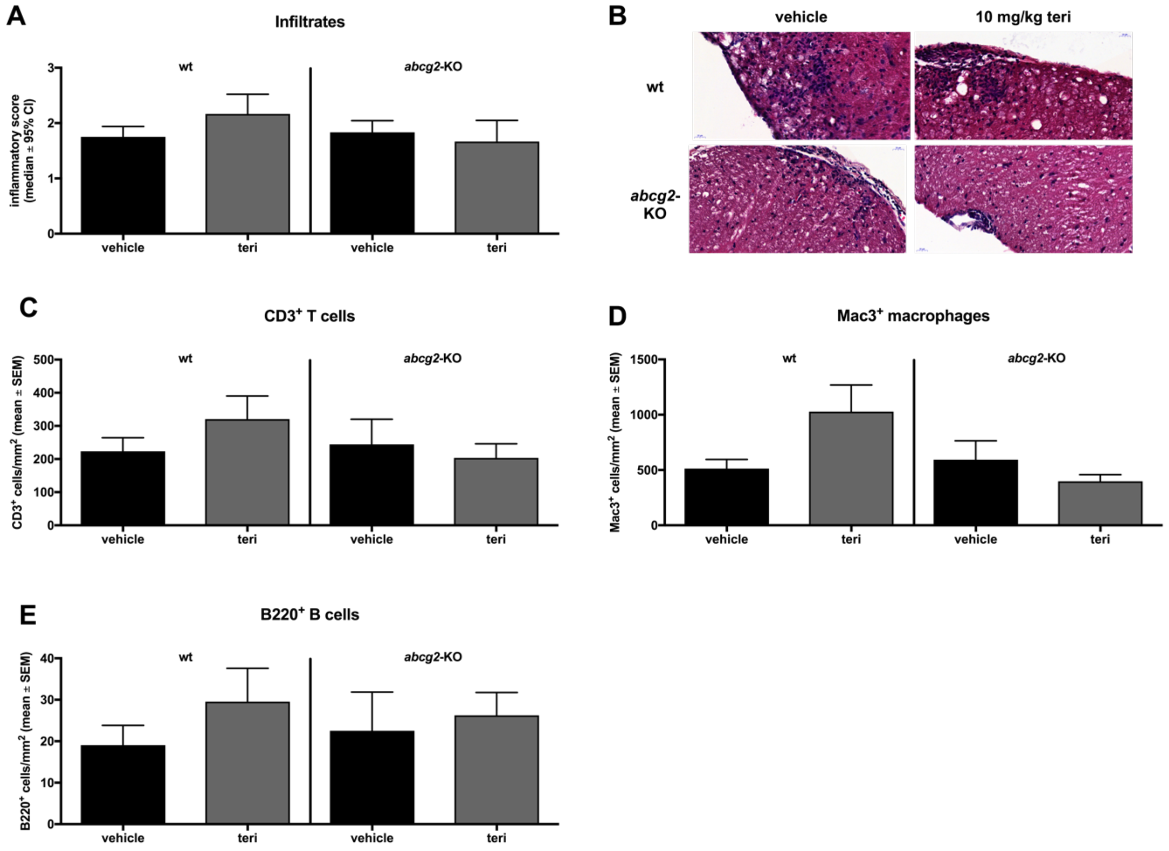

Supplementary Figure 3: Histolopathological characterization of spinal cords of *abcg2*-KO and wt mice after teri-treatment (10 mg/kg body weight, 17-20 days) during active MOG_{35-55} EAE. **(A)** Inflammatory score of spinal cord lesions (H&E staining); evaluated as inflammatory score as described in the methods, obtained at two sections of spinal cord tissue per each mouse. **(B)** Representative pictures of H&E staining (40x magnification, scale bar 20 µm). Quantification of **(C)** CD3+ cells (T-cells), **(D)** Mac3+ cells (macrophages) and **(E)** B220+ cells (B-cells); evaluated as cells/mm^2, obtained at two sections of spinal cord tissue per each mouse. A-D: n=6-13; MWU-test, p=ns. wt: C57BL/6J wild type mice; *abcg2*-KO: *abcg2*-deficient mice on C57BL/6J background; teri: teriflunomide.
