# Supplementary material for: Functional relevance of the multi-drug transporter abcg2 on teriflunomide therapy in an animal model of multiple sclerosis
Source: J Neuroinflammation. 2020 Jan 8;17:9. doi: 10.1186/s12974-019-1677-z (PMC6951012; doi:10.1186/s12974-019-1677-z)
Supplement: Supplementary file 4 — Additional file 4: Figure S4. Immunomodulation of T cell responses after teri-treatment during MOG35-55 EAE: Cytokine expression CD4+CD45+ and CD8+CD45+ T cells from spleen (A-D) and inguinal lymphnodes (E-H) and percentage of CD25+FoxP3+ of CD4+ T cells from spleen and inguinal lymphnodes (I-J). Teri-treatment (10 mg/kg) of active MOG35-55 EAE, once daily p.o. (4±1 days), individually after disease onset (Score>1) in abcg2-KO and wt mice. (A, E) Gating strategy to identify CD4+CD45+ and CD8+CD45+ T cells. (B, F) Percentage fraction of CD4+CD45+ and CD8+CD45+ cells. Cytokine expression of CD4+ T cells (C, G) and of CD8+ T cells (D, H). (I) Gating strategy to identify CD25+FoxP3+ cells of CD4+ cells. (J) Percentage portion of CD4+CD25+FoxP3+ cells. Data were assessed by flow cytometry. Two-Way ANOVA (Turkey’s multiple comparison test): *p<0.05; **p<0.01. wt: C57BL/6 J wild type mice; abcg2-KO: abcg2-deficient mice on C57BL/6 J background; teri: teriflunomide; LN: inguinal lymphnodes. [file 12974_2019_1677_MOESM4_ESM.pdf]

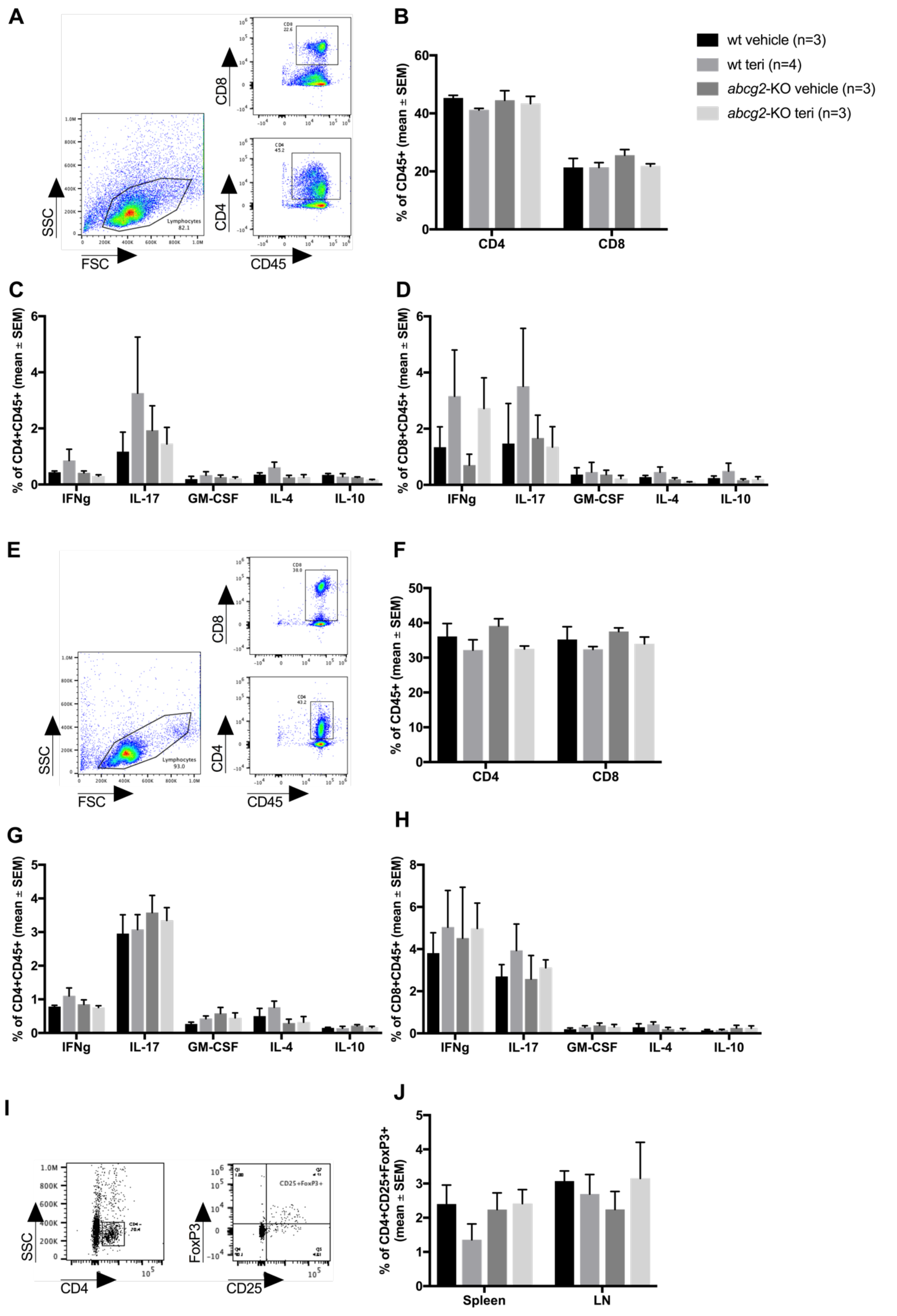
A
SSC
FSC
Lymphocytes 82.1
CD8
CD4
CD45
B
wt vehicle (n=3)
wt teri (n=4)
abcg2-KO vehicle (n=3)
abcg2-KO teri (n=3)
% of CD45+ (mean ± SEM)
CD4
CD8
C
% of CD4+CD45+ (mean ± SEM)
IFNg
IL-17
GM-CSF
IL-4
IL-10
D
% of CD8+CD45+ (mean ± SEM)
IFNg
IL-17
GM-CSF
IL-4
IL-10
E
SSC
FSC
Lymphocytes 93.0
CD8
CD4
CD45
F
% of CD45+ (mean ± SEM)
CD4
CD8
G
% of CD4+CD45+ (mean ± SEM)
IFNg
IL-17
GM-CSF
IL-4
IL-10
H
% of CD8+CD45+ (mean ± SEM)
IFNg
IL-17
GM-CSF
IL-4
IL-10
I
SSC
CD4
FoxP3
CD25
CD25+FoxP3+
J
% of CD4+CD25+FoxP3+ (mean ± SEM)
Spleen
LN

Supplementary Figure 4: Immunomodulation of T cell responses after teri-treatment during MOG_{35-55} EAE: Cytokine expression $CD4^+CD45^+$ and $CD8^+CD45^+$ T cells from spleen **(A-D)** and inguinal lymphnodes **(E-H)** and percentage of $CD25^+FoxP3^+$ of $CD4^+$ T cells from spleen and inguinal lymphnodes **(I-J)**. Teri-treatment (10 mg/kg) of active MOG_{35-55} EAE, once daily p.o. (4$\pm$1 days), individually after disease onset (Score>1) in *abcg2*-KO and wt mice. **(A, E)** Gating strategy to identify $CD4^+CD45^+$ and $CD8^+CD45^+$ T cells. **(B, F)** Percentage fraction of $CD4^+CD45^+$ and $CD8^+CD45^+$ cells. Cytokine expression of $CD4^+$ T cells **(C, G)** and of $CD8^+$ T cells **(D, H)**. **(I)** Gating strategy to identify $CD25^+FoxP3^+$ cells of $CD4^+$ cells. **(J)** Percentage portion of $CD4^+CD25^+FoxP3^+$ cells. Data were assessed by flow cytometry. Two-Way ANOVA (Turkey´s multiple comparison test): *p<0.05; **p<0.01. wt: C57BL/6J wild type mice; *abcg2*-KO: *abcg2*-deficient mice on C57BL/6J background; teri: teriflunomide; LN: inguinal lymphnodes.
