# Supplementary material for: Functional relevance of the multi-drug transporter abcg2 on teriflunomide therapy in an animal model of multiple sclerosis
Source: J Neuroinflammation. 2020 Jan 8;17:9. doi: 10.1186/s12974-019-1677-z (PMC6951012; doi:10.1186/s12974-019-1677-z)
Supplement: Supplementary file 5 — Additional file 5: Figure S5. Gating strategies of proliferation and apoptosis assays (see Fig. 1). (A) Gating strategy to identify the fraction of proliferating CFSE+PI- T cells. Representative figures of wt T cells treated with DMSO or teri (100 μM teri) for 48 h. Proliferation index was calculated as follows: normalized to ∅ cell death ( $=\% Proliferation\times \frac{\left(100-\varnothing cell\ death\right)}{100}$=%Proliferation×100−∅cell death100 ); quotient vehicle-treated cells to teri-treated cells ($=\frac{\% proliferation\ (vehicle)}{\% proliferation\ (teri)}$=%proliferationvehicle%proliferationteri x (-1)). (B) Gating strategy of apoptosis to identify percentage fraction of apoptotic T cells. Representative picutres of wt cells treated with DMSO for 48 h. Apoptosis was calculated as sum of Anx+, AnxPI+ and PI+ T cells. [file 12974_2019_1677_MOESM5_ESM.pdf]

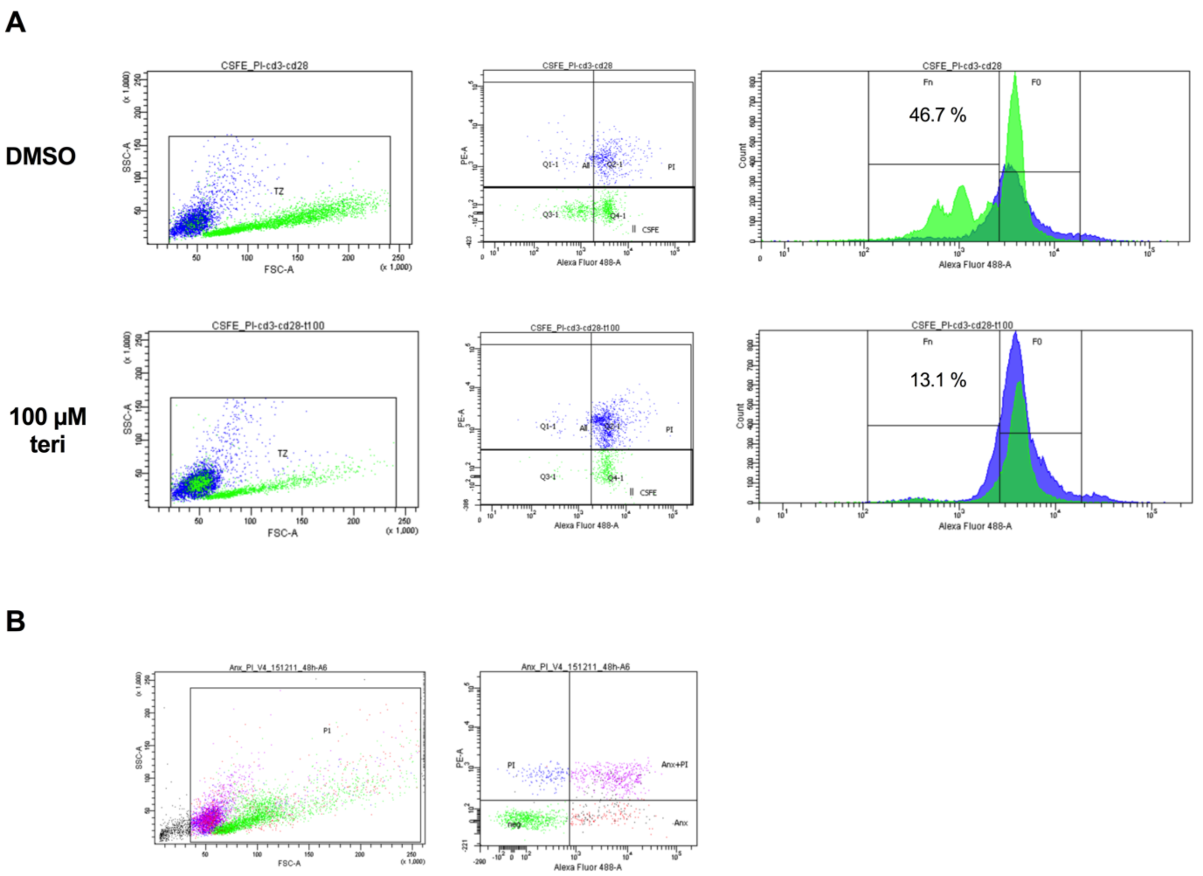

Supplementary Figure 5: Gating strategies of proliferation and apoptosis assays (see Fig. 1).

(A) Gating strategy to identify the fraction of proliferating CFSE$^+$PI$^-$ T cells. Representative figures of wt T cells treated with DMSO or teri (100 µM teri) for 48h. Proliferation index was calculated as follows: normalized to ∅ cell death ($= \% \, Proliferation \times \frac{(100-\emptyset \, cell \, death)}{100}$); quotient vehicle-treated cells to teri-treated cells ($= \frac{\% \, proliferation \, (vehicle)}{\% \, proliferation \, (teri)}$ x (-1)). **(B)** Gating strategy of apoptosis to identify percentage fraction of apoptotic T cells. Representative picutres of wt cells treated with DMSO for 48h. Apoptosis was calculated as sum of Anx$^+$, AnxPI$^+$ and PI$^+$ T cells.
