# Supplementary material for: Functional relevance of the multi-drug transporter abcg2 on teriflunomide therapy in an animal model of multiple sclerosis
Source: J Neuroinflammation. 2020 Jan 8;17:9. doi: 10.1186/s12974-019-1677-z (PMC6951012; doi:10.1186/s12974-019-1677-z)
Supplement: Supplementary file 6 — Additional file 6: Table S1. Flow cytometry antibodies. [file 12974_2019_1677_MOESM6_ESM.pdf]

Supplementary Table 1: Flow cytometry antibodies.

antigen	antibody-clone	isotype	fluorochrome	company
**CD45**	30-F11	Rat IgG2b	PE-Cy7	Biolgedend
**CD4**	GK 1.5	Rat IgG2b	Alexa700	Biolegend
**CD8**	53-6.7	Rat IgG2a	FITC	Biolegend
**IFN-γ**	XMG1.2	Rat IgG1	eF450	BD Biosciences
**IFNg**	XMG1.2	Rat IgG1	BrilliantViolet	BD Biosciences
**IL-17**	TC11-18H10.1	Rat IgG2a	eF610	BD Biosciences
**GM-CSF**	MP1-22E9	Rat IgG2a	PerCP/Cy5.5	Biolegend
**IL-4**	11B11	Rat IgG1	APC	eBioscience
**IL-10**	JES5-16E3	Rat IgG1	PE	Biolegend
